# Fluid Extracts

**Published:** 1868-10-01

**Authors:** 


### FLUID EXTRACTS.

Mr. Editor: A short time since there appeared in your columns, an article on the subject of "Fluid Extracts," signed H. D. Garrison. As reference was made several times to my process, and more particularly as said process was not in one instance correctly stated, allow me to disabuse the minds of your readers of the false impressions his article may have conveyed.

Mr. Garrison says: "The extracts to which you refer ('Duffields'') are patented, which, as before remarked, effectually patents the entire list." This view of the case, though worthy of serious consideration, is not the chief point to which I wish to direct special attention. Dr. Duffield's process *I believe consists essentially in producing a vacuum in his percolation and in his receiving vessel*, and then allowing the menstruum to flow in upon the drug, where he gets a pressure upon *his percolating menstruum* of nearly fifteen pounds to the square inch. *This rapidly filtering a liquid* through a drug, if not new, as some broadly intimate, is certainly expeditious, which I believe is its chief merit. That it avoids heat (whether judiciously or not) is *the best offered the profession.*"

The *italicizing* is mine, Mr. Editor.

Without entering into particulars, however much excused I might be, in the face of Mr. G.'s false assertions, allow me to take up the subject and prove two points:

1st. Mr. G. has not comprehended the process which he so virulently attacks, attributing, as he does, to its author "profound ignorance" or "unworthy motives," and,

2nd. I have not violated the principles of the United States Pharmacopœia.

Before "percolation" was adopted into the processes of the U. S. P., the plan of *maceration* was solely used, and in the edition prior to the one of 1866, (1858) while suggesting a *trial*

of percolation and giving both methods, it advised beginners and not thoroughly educated pharmaceutists, to hold preferably to maceration. I agree with Mr. Garrison that percolation when *rightly conducted*, will produce fluid extracts which contain the active principles perfectly preserved. The Pharmacopœia is the "hand-book" of the practical pharmaceutist, but can not be followed *verbatim* by the large manufacturer.

Mr. Garrison contends that "Dr. Duffield says his process removes the air and allows the alcohol to permeate the drug more perfectly." He attacks the process as *one of percolation*, because I made use of the word "permeate." Any one of your readers will understand the differences.

Mr. G. also contends that a vacuum *produces decomposition* of volatile bodies, *i. e.*, "alkaloids, ethers, oils, etc." Should these conclusions be proven correct, (which I claim can not be, unless chemical principles and affinities are upset completely), he will be individualized as the foremost in the rank of original investigators in this realm of organic chemistry. I admit an ether can be volatilized in vacuo, but I deny it is *decomposed* by simple heat in vacuo. Mr. Garrison has confounded *evaporation* and *distillation* with *decomposition*. Mr. Star, the famous toxicologist, recommends the use of a vacuum in evaporating ether from solutions of the alkaloids separated in analyzing the viscera, etc. Mr. Boudault prepares his famous "pepsin" in vacuo *to avoid decomposition*. *Sugar* must be made in vacuo to obtain it pure and nice. Mr. Garrison's idea is at direct variance with the views of our most accomplished zoöchemists, such as Liebig, Mulden, Star, Loing, Pasteur, and others. In order to promote decomposition we must have oxygen present, which is impossible in vacuo.

Pasteur's researches have demonstrated that oxygen is the *pabulum* of the vibriones and zoö-germs which promote decompositions. How, then, can decomposition set in in a vacuum which, being empty, can not contain the elements which support the existence of the organic spores which produce decomposition. Take the familiar act of canning fruit, and try and reconcile Mr. G.'s argument with the practical fact that *you do keep fruit better in a vacuum.*

Mr. Garrison has, in his attempt to prove that a vacuum is a promoter of decomposition, shown himself sadly deficient of the very first principles of organic chemistry, and made himself the laughing stock of every educated chemist. When writing a scientific article, it would be well to use the right terms, and not confound "evaporation" with "decomposition," nor "permeate" with "percolate."

I have studiously avoided attacking the Pharmacopœia processes, knowing that those processes were based upon years of experience. The principles I follow are the same as laid down in that much abused book. It has laid out formulæ based upon one pound of drug, on a scale commensurate with the wants of the dispensing chemist, should he see fit to become his own factor. It tells you to manufacture ether and spirits of nitre in glass retorts; but if I should use a large still made of lead, and operate a little differently to distill 100 pounds per day, I do not consider I have violated the Pharmacopœia, provided I produce the *same article* it requires. The hypercritical eye of Mr. Garrison views it differently. I claim the committee who compiled the U. S. P. had no such idea. They wished simply to have a standard that the pharmacist who makes his own preparations, could follow. It lays down *principles* — *Principia non formulæ* — and any one violating those principles should be arraigned. I use the same menstruum *macerating in vacuo* as long as I want to, from six to twelve days, as the case may require. It is one thing to percolate two pounds of drug, and another to percolate four hundred pounds. In one case there is hardly any chance of failure, in the other every chance of one.

